# Spatial Cognition and Range Use in Free-Range Laying Hens

**DOI:** 10.3390/ani8020026

**Published:** 2018-02-08

**Authors:** Dana L. M. Campbell, Andrew C. Talk, Ziyang A. Loh, Tim R. Dyall, Caroline Lee

**Affiliations:** 1 Commonwealth Scientific and Industrial Research Organisation (CSIRO), Agriculture and Food, Armidale, NSW 2350, Australia; tim.dyall@csiro.au (T.R.D.); caroline.lee@csiro.au (C.L.); 2 Adjunct to School of Environmental and Rural Science, University of New England, Armidale, NSW 2351, Australia; 3 School of Psychology, University of New England, Armidale, NSW 2351, Australia; atalk@une.edu.au; 4 Faculty of Veterinary and Agricultural Sciences, University of Melbourne, Parkville, VIC 3010, Australia; zloh@student.unimelb.edu.au

**Keywords:** T-maze, learning, radio-frequency identification, early enrichment, brain, fear

## Abstract

**Simple Summary:**

Individual free-range laying hens vary in their use of the outdoor range. The outdoor environment is typically more complex and variable than indoor housing and thus range use may be related to differences in spatial abilities. Individual adult hens that never went outside were slower to learn a T-maze task—which requires birds to repeatedly find a food reward in one arm of the maze, compared to outdoor-preferring hens. Pullets that were faster to learn the maze also showed more visits to the range in their first month of range access but only in one of two tested groups. Early enrichment improved learning of the maze but only when the birds were tested before onset of lay. Fear may play a role in inhibiting bird’s spatial learning and their range use. More studies of different enriched rearing treatments and their impacts on fear and learning would be needed to confirm these findings. Overall, these results contribute to our understanding of why some birds choose to never access the outdoor range area.

**Abstract:**

Radio-frequency identification tracking shows individual free-range laying hens vary in range use, with some never going outdoors. The range is typically more environmentally complex, requiring navigation to return to the indoor resources. Outdoor-preferring hens may have improved spatial abilities compared to indoor-preferring hens. Experiment 1 tested 32 adult ISA Brown hens in a T-maze learning task that showed exclusively-indoor birds were slowest to reach the learning success criterion (*p* < 0.05). Experiment 2 tested 117 pullets from enriched or non-enriched early rearing treatments (1 pen replicate per treatment) in the same maze at 15–16 or 17–18 weeks. Enriched birds reached learning success criterion faster at 15–16 weeks (*p* < 0.05) but not at 17–18 weeks (*p* > 0.05), the age that coincided with the onset of lay. Enriched birds that were faster to learn the maze task showed more range visits in the first 4 weeks of range access. Enriched and non-enriched birds showed no differences in telencephalon or hippocampal volume (*p* > 0.05). Fear may reduce spatial abilities but further testing with more pen replicates per early rearing treatments would improve our understanding of the relationship between spatial cognitive abilities and range use.

## 1. Introduction

Globally, the laying hen industry is transitioning away from conventional caged housing to alternative systems such as aviary, barn, furnished, and free-range. Outdoor range access is perceived to improve hen welfare [1,2]; thus, across Australia, free-range production is increasing. However, radio-frequency identification tracking of individual laying hens from both within Australia and internationally is consistently showing that within a flock, not every hen chooses to access the range every day, with some hens never venturing outside [3,4,5]. Behavioural assessments such as tonic immobility tests, manual restraint tests with plasma corticosterone measures, and open field tests have been used to reveal indoor-preferring, and exclusively-indoor hens present higher fear or stress reactions than outdoor-preferring hens [6,7,8]. The exclusively-indoor birds used specifically in this study were slower to move in an open field test compared to outdoor-preferring birds (*p* = 0.02) but did not differ in latency to vocalise, number of steps or number of vocalisations in the open field test, or in duration of tonic immobility (all *p* ≥ 0.08). A full report of these behavioural tests in a larger set of birds additional to the birds in this study is presented in Campbell et al. 2016. However, there is limited evidence to date of any differences in cognitive abilities between birds that vary in their range use patterns.

Chicks provided with short-term outdoor access have shown improved learning in a Y-maze task [9], and hens reared in more environmentally complex aviary systems had improved levels of working memory in a spatial holeboard task compared to birds reared in conventional cage systems [10]. These studies indicate spatial learning ability in laying hens can be experience-dependent and improved by environmental complexity during development. Different housing environments can also result in variation in hen brains such as in the hippocampus and nidopallium caudolaterale [11], but not in all cases, and thus may depend on extent of exposure to different housing environments and bird age [12]. The outdoor conditions in a free-range system are typically more dynamic compared to environmentally controlled indoor housing. Ranges often have large open spaces and varying topography, with navigation required to successfully return to the food, water, nestbox, perch, and shelter resources located inside. Given the differing levels of environmental complexity and/or environmental variation experienced by birds that vary in frequency of range access, one might expect differing spatial cognitive abilities. Birds that transition between indoor and outdoor areas may have improved spatial cognition compared to birds that remain indoors only. Differences in cognitive abilities could either be pre-existing in hens that choose the inside or outside environment, or develop as a result of experience with the more spatially complex outside range [9].

This research aimed to determine if differences in cognitive abilities may contribute to the extreme variation seen in individual ranging patterns for free-range hens and whether early rearing environments modify cognitive performance. Experiment 1 first retrospectively tested whether range use patterns correlated with performance in a T-maze learning task in adult free-range hens in which the outdoor-preferring hens were predicted to show faster learning rates and fewer errors. To isolate the effect of range access itself on spatial performance, Experiment 2 was conducted to assess learning rates in the same T-maze task using pullets prior to their first range access. It was predicted that the faster-learning pullets that made fewer errors would use the range more when first provided access. These birds had also been reared in two treatment groups to assess impacts of early enrichment on adult behaviour and welfare as part of a larger experiment [13]. It was predicted that the enriched-reared birds would perform better in the T-maze learning task. Finally, a subset of birds from the enriched and non-enriched treatment groups were also assessed for differences in hippocampal and telencephalon size where it was predicted the enriched birds would have larger capacity in these areas.

## 2. Materials and Methods

### 2.1. Ethical Statement

All research was approved by the University of New England Animal Ethics Committee (Experiment 1: AEC 15-129, Experiment 2: AEC 15-119) and complied with the Australian Code for the Care and Use of Animals for Scientific Purposes [14].

### 2.2. Experiment 1: T-Maze Learning in Adult Hens

#### 2.2.1. Animals and Housing

Test subjects were 31 ISA Brown laying hens selected from a previous study on the impacts of outdoor stocking density on range use [3]. In this previous experiment, the daily range visits of 450 individual hens that were housed in 6 pens of 150 birds per pen (50% of total 900 birds tagged) were tracked from 22 to 36 weeks of age using radio-frequency identification (RFID) technology. The raw RFID data were filtered through a custom-designed software program that removed ‘false’ unpaired readings (Bryce Little, CSIRO, Agriculture and Food, St Lucia, QLD, Australia). The program then summarised the percentage of available ranging days that hens accessed the outdoors (for any amount of time on each day). Individual hens varied in how often they visited the range, with the majority of birds accessing the range on 100% of available days. However, a small proportion accessed the range on less than 10% of available days and a small proportion never went outside [3].

Birds from all range use categories were represented within each outdoor stocking density treatment; thus, focal hens for the current study were selected from all experimental pens. Seventeen hens were selected that chose to visit the range on 100% of available days (outdoor-preferring hens), 8 hens that chose to visit the range on 2–10% of available days (indoor-preferring hens), and 7 hens that never went outside (exclusively-indoor hens). These different sample sizes per group were proportional representations of the ranging categories. All hens were in visibly healthy physical condition, with no feather pecking as shown by individual assessments conducted at monthly intervals throughout the range tracking period as part of a separate dataset [15]. Between weeks 36 and 38, subject hens from the current study (including additional focal birds as the once-only tests were able to be completed on a larger number of birds) were assessed in tonic immobility, manual restraint, and open field tests as part of a separate dataset [6]; hens were returned to their original pens after daily testing.

At 38 weeks of age the selected hens for this study of T-maze learning were grouped together into one indoor pen at the University of New England’s Laureldale Poultry Facility (Armidale, NSW, Australia) with indoor food, water, perch, and nestbox resources either set to or exceeding the Australian Model Code of Practice for the Welfare of Animals—Domestic Poultry [16]. Management practices remained as prior following the re-grouping into a single pen. During experimental days, hens were caught in no specific order from the home pen and then placed into a second temporary pen (with perches, water and food) following testing to avoid re-catching the tested birds. All hens were then returned to the home pen after testing concluded each day and were provided range access via pop holes for the evening. All birds had range access from 09:00 to 19:00 on non-testing days. Range use was not tracked following relocation to the new pen, but individual range use patterns had been consistent over the 15-week tracking period prior to focal bird selection. Birds were tested from 49 to 52 weeks of age.

#### 2.2.2. T-Maze Habituation

All 32 birds were exposed to live mealworms prior to the maze testing, as these were a novel food reward. Each bird was carried to an experimenter and gently held with feet on the ground while being presented a cup of mixed grain (not novel to the hens) with mealworms placed on top. The ISA Brown adult hens are typically not reactive to people and will readily approach rather than avoid personnel. Each bird ingested 3 mealworms before being relocated to the post-testing pen. During all tests, all experimenters were blind to the ranging status of the birds.

**Figure 1:**
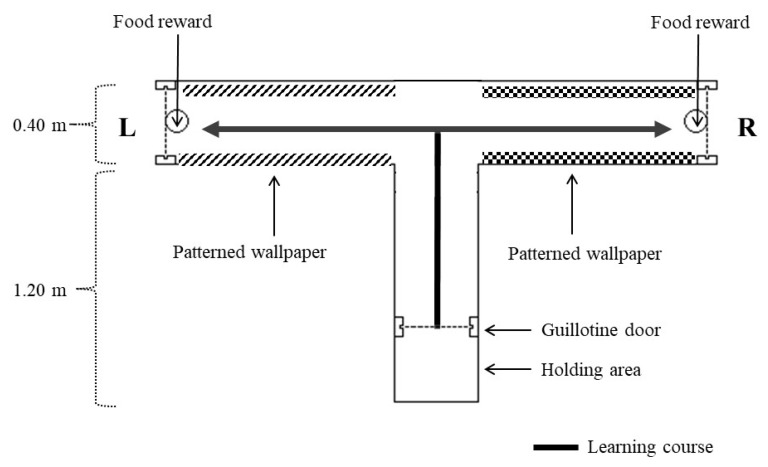
A top-down schematic of the T-maze apparatus showing placement of the food reward (alternating placement at L or R side), patterned wallpaper in the L and R arms, and the learning courses with a guillotine door for keeping birds in the holding area. All maze walls were 0.80 m in height.

A wooden maze with 3 arms of equal length was placed outside the home pen, where test subjects were visually but not acoustically isolated from flock mates. The placement of the maze in this location minimised transport and handling of the hens. The maze was left in the same position for the duration of testing so all extramaze cues were consistent. The centre arm was designated as the arm to place hens into and contained a starting box with a raiseable guillotine door and the left/right arms were choice arms (Figure 1). The 2 arms were visually different and lined with contrasting wallpaper (black and white zig zags or polka dots) to provide intramaze cues, but both arms contained red-coloured food reward cups placed at the arms’ end (Figure 1). The food reward consisted of mash (the normal diet) with 3 mealworms placed on top, and the hen had to approach the cup to be able to see if it contained food. Each maze arm was covered with hinged wire mesh covers to prevent hen escape (but enable hen capture after testing) with all trials observed live via monitors connected to Sony HDR-XR260E video cameras placed over the arm ends allowing the experimenters to remain out of sight. All birds were handled by the same experimenter.

All hens (*n* = 32) were individually habituated to the T-maze over 4 days with a total of four 5 min sessions per hen. Each hen was placed in the starting box (Figure 1) for 30 s before the door was raised and the hen was allowed to explore the maze (both food reward cups were present but empty), noting whether she visited one or both arms to ensure experience with both maze arms. Criteria for habituation was that the hen was walking in the maze (i.e., not frozen) and had visited both arms of the maze. One indoor-preferring hen was excluded from testing due to repeated escape attempts during habituation, and an exclusively-indoor hen did not leave the start box (for 3 consecutive habituation sessions) and thus was also excluded.

#### 2.2.3. T-Maze Learning

Learning occurred over several days in a step-wise method. To increase motivation, *ad libitum* food was removed from the home pens the night prior. Food was restored *ad libitum* in the home pen immediately after daily testing and all birds had access to *ad libitum* food in the post-testing pen. A maze side arm (left or right) was randomly assigned for each bird to be trained on with the exception of one indoor-preferring hen, which displayed a strong side bias during habituation (noted to mostly visit one maze arm over the other), and thus, was allocated the opposite side. Each day consisted of 1 session per hen with 5 trials per session.

In the first training session (Day 0), the hen was placed in the starting box for 10 s. After the 10 s had elapsed, the door was lifted and the hen was allowed to explore the maze and find the reward in the allocated arm. Hens that were immobile for 20 s were encouraged to the correct direction by placing an A4 sized wooden board behind the bird to gently encourage forward movement. If the hen was unsuccessful in finding the correct arm in 2 min, she was gently directed to the correct arm using the board. After each trial, the hen was carried back to the starting box and the next trial would begin. Latency and error data from the first training session were not included in analyses.

Subsequent testing sessions (Day 1–7 with *n* = 30 birds; 17 outdoor-preferring, 7 indoor-preferring, 6 exclusively-indoor hens) were identical; however, encouragement after 20 s of immobility was not required, as all birds left the start box once the door was raised and were motivated to access the maze arms. Hens were still directed to the correct arm if they did not find the food reward within 2 min so they could learn the task [17]. In these testing sessions, hens were required to achieve a success rate of minimum 80% correct choices over 5 sessions (20 correct choices of 25 trials) and latency (seconds) to each success (from the time the door was raised until the bird reached the food reward and started to consume) was recorded.

Each success was defined as the hen consuming the food reward in the correct arm without having entered the incorrect arm prior. An unsuccessful trial was defined as either entering the incorrect arm and/or not finding the food reward within 2 min. If both feet had passed the entrance of the incorrect arm or the centre arm, it was considered a choice and the error was recorded. Following an error, the hen was still permitted to continue searching for the food reward within the allocated 2 min. If hens were unsuccessful in reaching criterion over the first 5 days, the first of the 5 days was not included for criterion selection and the second day would then become the first and so forth until criterion was reached. However, all days of testing were included in the data analyses. Hens that were successful in fulfilling the criterion were no longer tested.

#### 2.2.4. Data and Statistical Analyses

The number of encouragements and directing towards the food during the first training day were compared between range use groups using Kruskall-Wallis tests. Data from the T-maze learning days were compiled into the total latency (seconds) to reach 80% success criterion (summed time for each individual trial until minimum 80% success was achieved), and the total number of errors (visits to the incorrect or centre arm) made until minimum 80% success. Total latency to success and summed error rate data were square-root transformed. Differences between outdoor-preferring, indoor-preferring, and exclusively-indoor hens were compared using general linear models with post-hoc Student’s *t*-tests applied to the least squares means. The raw values are presented in the Results, as there were no differences between the raw and back-transformed means. All analyses were conducted in JMP^®^ 13.0.0 (SAS Institute, Cary, NC, USA) with α set at 0.05.

### 2.3. Experiment 2: Early Enrichment and T-Maze Learning in Pullets

#### 2.3.1. Chick Housing and Early Enrichment

Day-old infrared beak-trimmed Hy-Line Brown chicks (*n* = 315) were obtained from a commercial supplier and randomly allocated into two separate rooms (4.5 mL × 3 mW) at the University of New England Animal House facilities where they were housed until 12 weeks of age. There was only one room per treatment group due to housing facility limitations. These birds were part of a larger experiment [13] with only 117 birds used for the current study. Temperature control and hours of lighting followed the recommended management guidelines [18]. Commercial mash for specific growth stages was provided *ad libitum* with access to water nipples (10 birds/nipple) and wood shavings as a floor substrate. At 4 weeks of age, one perch rack (1.6 mH × 2.2 mW with 6 perch bars) per room was supplied.

Birds in one room (standard non-enriched conditions) had no additional interventions, with personnel entering the room approximately once per day after week one. Birds in the second room (enriched treatment) were subjected to a novel, changing, unpredictable environment from 4 to 21 days of age. A variety of stimulation was provided, including patterned wallpaper, cinder blocks, large sealed plastic tubs, novel objects attached to feeders and water nipples, coloured flashing lights, and auditory playbacks that included sounds of doors opening, moving vehicles, weather, voices, and machinery. Enrichments were exchanged (by the same experimenter) on a daily basis or moved within the pen, with lights and playbacks on a random schedule. This changing enrichment schedule was hypothesised to make birds more adaptable to environmental stress and change. No adverse reactions to the stimuli (e.g., panic) were anecdotally observed. After 3 weeks of age, all birds in both rooms were housed in the same conditions.

#### 2.3.2. Pullet and Layer Housing

At 12 weeks of age all birds were moved to the University of New England’s Laureldale Poultry Facility and distributed between 6 pens (3 enriched rearing treatment, 3 non-enriched rearing treatment). Only 2 pens of birds (1 enriched, 1 non-enriched) were used for further testing in this study. All pens had equal food, water, perch, and nestbox resources that exceeded the Australian Model Code of Practice for the Welfare of Animals—Domestic Poultry [16]. Indoor stocking density was approximately 3 birds per m^2^, with rice hulls provided as a litter substrate. Birds were fed *ad libitum* commercial mashes formulated for pullet followed by layer life stages. The birds were destined for free-range access, but this was not provided until 21 weeks of age. Further details on the pen layout and shed are available in Campbell et al. 2017b [13].

#### 2.3.3. Test Subjects and T-Maze Habituation

Two pens of birds (enriched pen *n* = 57 birds, non-enriched pen *n* = 60 birds) were used for training in the same T-maze as used in Experiment 1. Due to the time required to train birds in the T-maze, limited numbers of birds could be tested. Additionally, across the 6 groups of birds, small numbers were predicted to be indoor-preferring or exclusively-indoor hens. RFID tracking with previous flocks showed typically small percentages of indoor birds per pen, but similar patterns across all pens [3]. Thus, all birds within a single pen were tested rather than samples of birds from all pens to ensure a greater chance of having T-maze data on birds that would vary in their range use. The maze was again placed outside the home pens, visually but not acoustically isolating test subjects from flock mates. The maze remained in the same position for testing duration. All trials were again observed live, via monitors connected to Sony HDR-XR260E video cameras, thus allowing the experimenters to remain out of sight. All experimenters were blind to the rearing status of the birds.

All test birds were initially exposed to the novel live mealworms in their home pens (placed in large floor pans) and allowed to consume them *ad libitum*. Due to the large number of birds being trained in the T-maze, birds were tested in two age groups across an approximately 2-week period each (Age group 1: 15 to 16 weeks of age, *n* = 29 enriched, *n* = 28 non-enriched; Age group 2: 17 to 18 weeks of age when some birds were starting to come into lay, *n* = 28 enriched, *n* = 32 non-enriched).

Prior to each habituation, training, or testing day, all subject hens from one age group/treatment (e.g., Age group 1, enriched birds) were caught from the home pen and placed into a temporary holding pen (of the same size with water access and nestboxes) and permitted to settle for 15 min. Hens were first habituated to the T-maze in groups of either 4 or 3 birds (to socially encourage maze exploration), with a total of two 15 to 17 min sessions per hen group across 2 separate days. Each hen was placed in the starting box for 30 s before the door was raised and the hen was allowed to explore the maze (both food reward cups were present but empty). A second hen was immediately placed into the starting box for 30 s and so forth until all 4 (or all 3) hens were in the maze simultaneously. After 15 min of habituation (timed from the release of the last hen in the group), hens were returned to their home pen and the next set of birds were placed into the maze. All hens were observed via the monitors to ensure all birds had visited both arms of the maze.

#### 2.3.4. T-Maze Learning

Learning to access the food reward in the T-maze arm occurred over several days in a step-wise method similar to the protocol used in Experiment 1. Birds were food-restricted overnight prior to daily trials to increase motivation, with food immediately restored in the home pen after testing. Birds were caught in no specific order from the temporary pen, placed into the start box individually (for 10 s), then provided access to the maze. The side placement of the food reward for each bird was chosen by allocating the opposite side of the maze to the first side they chose to enter (thus avoiding training to a possible side bias). Immediately following their first side choice, birds were caught and returned to the start box to begin training with the food reward now placed on the opposite side. Each day then consisted of one session per hen with 5 trials per session; the same protocol of holding in the start box for 10 s was used for all trials.

In the first training session (Day 0), after the starting box door was raised, the hen was allowed to explore the maze and find the reward in the allocated arm. If the hen was unsuccessful in finding the correct arm within 2 min, she was gently directed to the correct arm using a wooden clipboard and allowed to eat the food reward. After each trial, the hen was gently directed back to the starting box (to avoid excessive bird handling) and the next trial would begin. Subsequent testing sessions were identical, with all trials allocated a maximum of 2 min to find the food reward. The same criterion was used as per Experiment 1 in which hens had to achieve minimum 80% correct choices over 5 sessions (20 correct choices of 25 trials). The latency to each correct choice including number of errors made was recorded. Hens that reached the success criterion were no longer tested. All birds in Age group 1 were tested until minimum 80% success criterion, then all birds in Age group 2 were habituated and tested until minimum 80% success criterion. The testing order of enriched versus non-enriched birds within each age group was balanced across testing days.

#### 2.3.5. Brain Histology

At 21 weeks of age, a randomly-selected subset of hens (*n* = 24) across both treatment groups (12 hens per treatment) were given an overdose of Na-Pentobarbital and perfused transcardially with 0.8% saline followed by 10% formalin in 0.8% saline. Their brains were removed from their skulls and allowed to postfix for at least 7 days in 10% formalin/0.8% saline. The brains were then transferred to 10% formalin in 30% sucrose until they sank (3 to 4 days). For slicing, the brains were then embedded in Optimal Cutting Temperature compound and frozen in a freezing microtome to −20 °C. Coronal sections through the brain were taken at 50 μm and every second section through the hippocampus was plated onto a subbed microscope slide. The sections were allowed to dry on the slides, then stained with cresyl-violet, cleared with ethanol and histolene, and cover-slipped.

#### 2.3.6. Telencephalon and Hippocampal Formation Size Measurements

Digital images of the stained brain slices were obtained using a high-resolution slide scanner. For sections containing the anterior commissure, the outlines of the telencephalon and of the hippocampal formation (the hippocampus and medial and lateral parahippocampal areas; [19]) were traced using Adobe Photoshop^®^. The observer tracing the brain areas was blind to the treatment groups. Taking the measurements specifically from sections containing the anterior commissure assured that for each subject the area measurements were taken from sections at the same anterior/posterior level. The number of pixels within the traces were counted by Photoshop^®^ and converted to mm^2^ area measurements.

#### 2.3.7. Radio-Frequency Identification of Range Use

The two indoor test subject pens were associated with separate fenced, straight outdoor runs (3.7 mW × 32.1 mL) with no shade or shelter structures but with shade cloth placed along the fences to minimise visual contact between the ranges. All birds were fitted with a microchipped leg band to track their daily (09:00 to 16:30 h) range use for 4 weeks across autumn (sunset ~17:30 h) starting from 21 weeks of age. Natural range usage was assessed and birds were encouraged to return inside each afternoon using poultry grain mix. Birds were held inside at all other times. Further details on the RFID data collection equipment and procedures are available in Campbell et al. 2017b [13].

#### 2.3.8. Data and Statistical Analyses

The latency to success (seconds) and number of errors made in reaching success were compiled for Age group 1 and Age group 2, enriched and non-enriched birds, separately. The latency and count data were square-root transformed to approach normality. General linear models were used to compare the effects of rearing treatment, age group, and their interaction. Where significant differences were present, post-hoc Student’s *t*-tests were applied to the least squares means. Differences between enriched and non-enriched treatment groups in telencephalon and hippocampal size measurements were compared using two-tailed *t*-tests.

All raw daily RFID data were first filtered through the custom-designed software program that removed ‘false’ unpaired readings. The program then summarised the average daily hours spent outdoors and the average daily number of visits to the range for each individual bird. RFID and T-maze data were square-root transformed. Simple linear regressions were then applied to determine if the latencies to success and number of errors made during T-maze learning would predict average hours or average number of visits outside separately for enriched and non-enriched birds.

All analyses were conducted in JMP^®^ 13.0.0 (SAS Institute, Cary, NC, USA) with α set at 0.05. The raw values are displayed in the figures, as there were no differences between the raw and back-transformed data.

## 3. Results

### 3.1. Experiment 1: T-Maze Learning in Adult Hens

**Figure 2:**
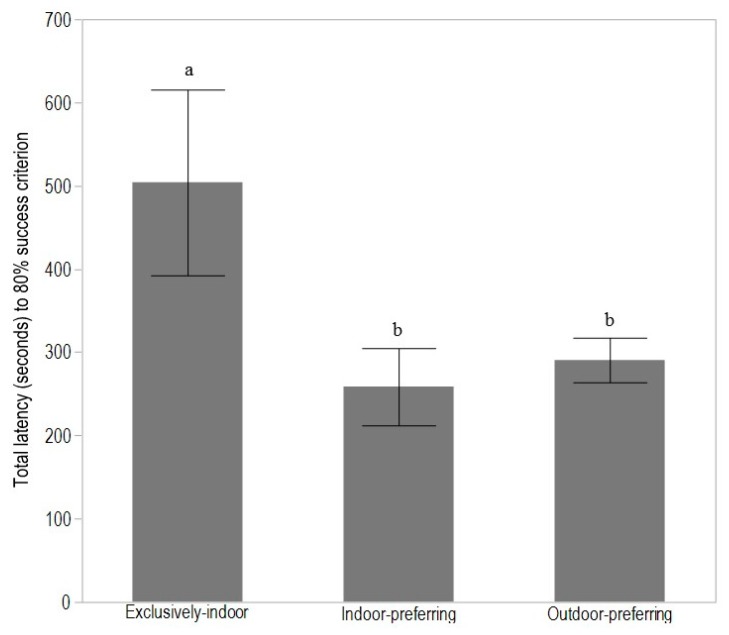
The mean ± SEM total latency (seconds) to minimum 80% success criterion during learning of the T-maze task for exclusively-indoor (range access on 0% of available days), indoor-preferring (range access on 2–10% of available days), and outdoor-preferring (range access on 100% of available days) adult hens. Different superscripts indicate significant differences across range access groups (*p* < 0.05).

There were no differences between range use groups in number of encouragements to move (*p* = 0.39), or in directions towards the feed during the first training day (*p* = 0.09). All hens reached criterion within 7 days of testing. There was an effect of range access group in total latency to reach the T-maze learning success criterion (F_(2,27)_ = 4.72, *p* = 0.02), with exclusively-indoor hens showing the longest latency to success (*p* < 0.05, Figure 2). However, there were no differences between range access groups in the summed error rate during learning (F_(2,27)_ = 1.52, *p* = 0.30; LSM ± SEM: outdoor-preferring 3.25 ± 0.51, indoor-preferring 2.43 ± 0.78, exclusively-indoor 3.83 ± 0.84).

### 3.2. Experiment 2: Early Enrichment and T-Maze Learning in Pullets

**Figure 3:**
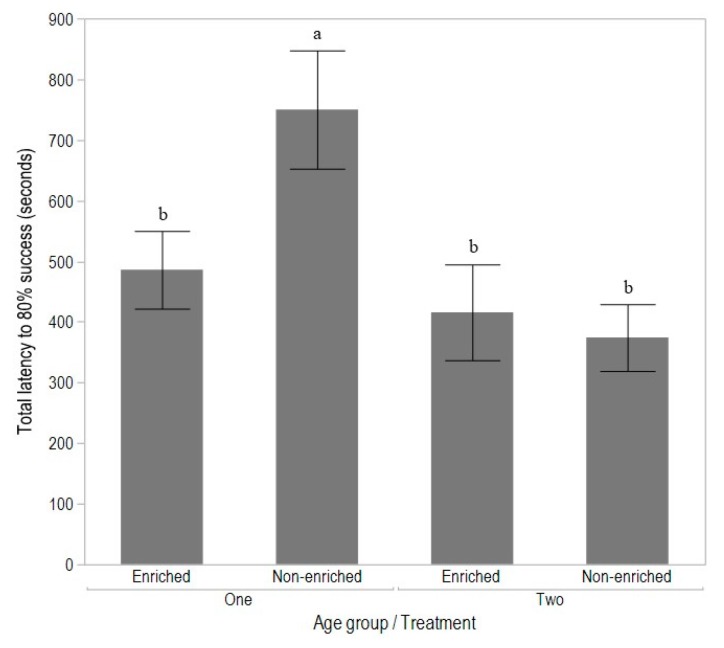
The total latency to minimum 80% success (seconds) during T-maze learning for birds from enriched or non-enriched early rearing treatments and tested as Age group 1 (15 to 16 weeks of age) or Age group 2 (17 to 18 weeks of age). Different superscripts indicate significant differences between treatments across testing age groups (*p* < 0.05).

The majority of birds (64% in Age group 1, 87% in Age group 2) reached criterion within the minimum time of 5 days, with some individuals taking up to 10 days. Three non-enriched and 2 enriched birds were excluded from the T-maze trials, as they were unable to reach criterion. There was a significant interaction between age group and treatment (F_(1,111)_ = 5.43, *p* = 0.02), with the non-enriched birds of Age group 1 showing the longest latency to reach success (*p* < 0.05), but there were no differences between treatments in Age group 2 (Figure 3).

**Figure 4:**
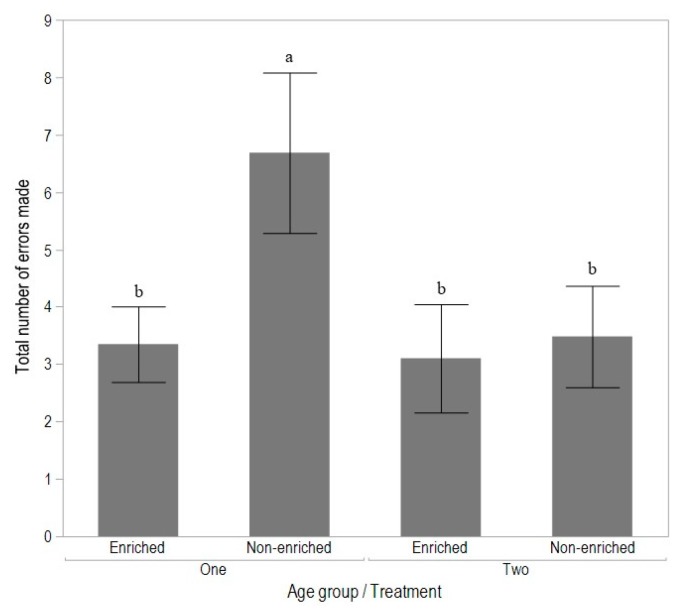
The total number of errors made in reaching the minimum 80% success criterion during T-maze learning for birds from enriched or non-enriched early rearing treatments and tested as Age group 1 (15 to 16 weeks of age) or Age group 2 (17 to 18 weeks of age). Different superscripts indicate significant differences between treatments across testing age groups (*p* < 0.05).

There was a significant interaction between age group and treatment in the number of errors made during the latency to reach success (F_(1,111)_ = 7.53, *p* = 0.01), with the non-enriched birds of Age group 1 showing the most errors (*p* < 0.05), but there were no differences between treatment groups in Age group 2 (Figure 4).

There were no differences between enriched and non-enriched birds in the size of the telencephalon (t_(20.21)_ = 0.18, *p* = 0.86, LSM ± SEM enriched birds: 54.74 ± 0.69 mm; non-enriched birds: 54.92 ± 0.69 mm) or hippocampus (t_(20.97)_ = 0.42, *p* = 0.68, LSM ± SEM enriched birds: 1.52 ± 0.15 mm; non-enriched birds: 1.61 ± 0.15 mm).

**Figure 5:**
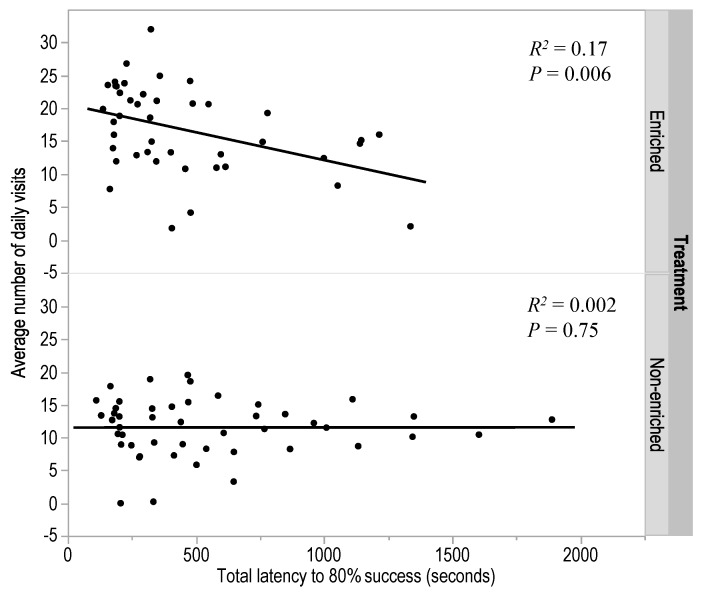
The regression between the total latency to reach the minimum 80% success criterion in T-maze learning and the average number of daily visits to the range for birds from enriched and non-enriched early rearing treatments.

There was a significant negative relationship between the latency to reach the minimum 80% success criterion and the average number of visits outside for the enriched birds (F_(1,41)_ = 8.35, *p* = 0.006, *R*^2^ = 0.17), but no relationship was present for the non-enriched birds (*p* = 0.75, Figure 5). Overall, the enriched birds also showed more visits to the range across the first 4 weeks (t_(−4.35)_ = 71.12, *p* < 0.0001; LSM ± SEM enriched birds: 16.75 ± 1.01 visits; non-enriched birds: 11.56 ± 0.63 visits). There were no relationships between the latency to reach minimum 80% success criterion and the average hours spent outside for enriched (*p* = 0.36) or non-enriched birds (*p* = 0.47). There was a trend for birds that made more errors to also show fewer visits to the range for the enriched birds (F_(1,41)_ = 3.21, *p* = 0.07, *R*^2^ = 0.07) but not for the non-enriched birds (*p* = 0.25). There were no relationships between number of errors made and the hours spent outside for enriched or non-enriched birds (all *p* ≥ 0.13).

## 4. Discussion

The two experiments conducted on free-range laying hens showed that performance in a spatial maze task had a relationship with use of the outdoor range. Retrospectively, birds that preferred to stay indoors were slower to learn the T-maze task. Maze learning rate also predicted future ranging behaviour in terms of number of daily range visits, but only in enriched birds. Maze performance was affected by early enrichment during rearing, but not at all tested ages. The enrichment treatment was limited to one pen per treatment for rearing, maze testing, and range use; thus, further study is needed to confirm the impact of rearing on spatial cognition and ranging.

In the first experiment, maze learning rate was retrospectively correlated with range access differences that were consistently displayed by hens during a ranging period of 15 weeks [3]; thus, causal relationships could not be determined. It is possible the exclusively-indoor hens had diminished spatial abilities as a result of individual variation resulting from genetic history or differential pre-natal environmental exposures [20], and this resulted in a preference to stay indoors. It is also possible that the choice to stay indoors when the pop holes were open, which may be based on higher levels of fear and stress [6,7,8], subsequently resulted in reduced spatial abilities at the later testing points. Range access of only one week resulted in enhanced spatial abilities during laboratory Y-maze tests in young laying hen chicks [9]. This significant impact of limited range exposure shown in chicks could account for the lack of differences between indoor-preferring and outdoor-preferring birds that had vastly different ranging strategies. These birds were also tested in the maze following transfer to a different pen within the same facility and their range use was not tracked in this new social environment. Thus, repetition of the maze test with a separate flock of birds is warranted to replicate these findings.

In contrast to Experiment 1, the second experiment that tested pullets prior to any range access was able to determine that differences in spatial abilities were pre-existing and could predict subsequent range use patterns. Specifically, birds that learned the maze faster showed higher numbers of visits to the range, although only in the enriched birds. This might suggest that these birds had greater spatial memory abilities that enabled them to be more confident and more exploratory; thus, they moved frequently between the indoor and outdoor areas. Food and water were located indoors, but more space and opportunity for behavioural expression such as foraging or dust bathing were available outdoors. Frequent transitions between the two areas in the free-range environment may optimise an individual’s health and welfare [21] and adaptation to provision of housing area choice. However, the welfare impact of frequent indoor/outdoor transitions versus single long periods outdoors needs to be investigated further.

The predictive relationship between maze learning rate and ranging was only present in the pen of enriched-reared birds. The rearing treatments in this study are limited, as only one rearing room was used per treatment group, but the differences in treatments were highly distinct. Furthermore, maze learning rate and ranging were only assessed in one pen from each treatment of the 3 treatment pens present at the free-range layer facility [13]. The use of only one pen per treatment was to ensure that maze data were captured from birds that would be likely to show variation in ranging. If samples of birds had been selected across multiple pens from each treatment, the chances of selecting low-ranging birds would have been highly reduced. Low percentages of birds typically choose to stay indoors within a single pen, but similar patterns are seen across all experimental pens [3,13]. However, there is some evidence from these results that the enriched-rearing treatment impacted spatial performance. Rearing treatment also impacted stress responses and adaptability to change in the same flock of birds later in the lay cycle [13]. The improved spatial ability of the enriched birds in the maze task was predicted, as environmental complexity during rearing has previously been shown to improve spatial performance of hens [10] and other bird species [22,23]. Thus, this rearing treatment may have enhanced pre-existing individual differences, resulting in the relationship between visit number and maze learning, compared to a lack of any relationship detected in the non-enriched birds.

Due to logistical time constraints involved in learning the maze, for Experiment 2, the birds (from both treatments) were tested in two different groups across time. Unexpectedly, there was a clear difference in maze learning rate and errors between the two ages, with the non-enriched birds performing equal to the enriched birds at the older age when birds were starting to come into lay. Anecdotally, this coincided with a distinct reduction in the general fear behaviour of all birds. Low and nil range users have previously been shown to have higher levels of fear and greater stress responses [7,8], including the birds used in this study (see Campbell et al. 2016 for a full report on behavioural tests, including additional focal birds to this study). Fear tests were not conducted on the birds in Experiment 2, but early enrichment similar to what was applied in this study has previously resulted in reduced fear responses in behavioural tests, albeit using a different strain of birds and different age of testing [24]. Similarly, the results of detour-tasks with young chicks searching for a disappearing social partner or mealworm prey suggest that fear of the novel testing environment affects the bird’s ability to complete both tasks more than specifically their cognitive abilities [25]. Correspondingly, fear levels may have impacted the hen’s learning responses to the T-maze across both experimental groups [26]. Habituation to the maze was designed to reduce the fear of the novel environment, but some birds may have still had higher underlying fear responses to the isolated testing arena that interfered with their learning. This interdependent relationship between fear and spatial cognition may be related to the differing hemispheric functions of the hen’s lateralised brain [27]. Hemispheric dominance may also be altered by hormonal changes [28], which could account for the reduced fear and faster maze learning in Age group 2 within Experiment 2. Distinct from a testing environment, the interplay between fear and spatial cognition could impact hen’s movement patterns in a commercial setting. Thus, early enrichment may be important for reducing fear and/or improving spatial abilities and thus optimising adjustment to layer housing following transfer from rearing facilities, which occurs just prior to onset of lay. The relationships between spatial performance, range access, and impacts of rearing environments on hemispheric dominance are avenues that warrant further investigation.

One cognitive test was conducted on these flocks of birds to investigate the relationship with differing range use patterns in which faster learning rate was used as an indicator of enhanced cognition. Faster learning rates in a laboratory-based associative learning task have been correlated with improved natural foraging success in bumble-bees [29]. Black-capped chickadees that were faster to learn an acoustic discrimination task also entered a novel environment more rapidly [30]. The T-maze test used in this study furthers our understanding of differences between outdoor and indoor-preferring birds in a free-range environment. However, further tests such as the spatial holeboard task [10], or a comprehensive test battery [31], would further determine if there are differences in general intelligence between birds that vary in range use, or if outdoor-preferring birds are, for example, more exploratory, impulsive, or goal-seeking.

Finally, there was no detection of predicted differences in telencephalon or hippocampal volume between the enriched and non-enriched birds when sampled at 21 weeks of age. The complexity of the enriched-rearing environment was expected to increase brain capacity where the avian hippocampus is involved with spatial learning and will increase in volume with increased use [32], and the avian telencephalon or forebrain is involved in cognition [33]. Adult laying hens (48 weeks of age) kept in different housing systems (battery cages, floor litter pens, and free-range) from 16 weeks onwards showed neuroanatomical differences, with free-range hens having larger cells in the dorsomedial hippocampus than caged but not floor-litter housed hens [11]. In contrast, neuroanatomical studies on a separate group of birds from the same adult environments showed no rearing-treatment impacts on the immunohistochemical detection of tyrosine hydroxylase in the hippocampus at 20 or 24 weeks of age [12]. Differences may have been detected in the current study if other measures were made such as dendritic length [34], neurogenesis [35], lateralisation, or immunohistochemical staining [12]. Alternatively, the enriched rearing treatment may not have been sufficient to have neurological impact.

## 5. Conclusions

This study indicates that range use is related to hens’ spatial abilities, which may account for differences in the high variation in individual use of outdoor areas. However, there is likely an interplay between fear and cognition, which might be modified via early rearing environments. Future study using a range of cognitive tests would further elucidate the differences between ranging and non-ranging birds. The enrichment treatment was limited to one pen per treatment for rearing, maze testing, and range use; thus, further study would be needed to confirm the impact of rearing on spatial cognition and ranging.
